# Microhomology Directs Diverse DNA Break Repair Pathways and Chromosomal Translocations

**DOI:** 10.1371/journal.pgen.1003026

**Published:** 2012-11-08

**Authors:** Diana D. Villarreal, Kihoon Lee, Angela Deem, Eun Yong Shim, Anna Malkova, Sang Eun Lee

**Affiliations:** 1 Department of Cellular and Structural Biology, University of Texas Health Science Center at San Antonio, San Antonio, Texas, United States of America; 2 Department of Molecular Medicine, Institute of Biotechnology, University of Texas Health Science Center at San Antonio, San Antonio, Texas, United States of America; 3 Department of Biology, School of Science, Indiana University–Purdue University Indianapolis, Indianapolis, Indiana, United States of America; National Cancer Institute, United States of America

## Abstract

Chromosomal structural change triggers carcinogenesis and the formation of other genetic diseases. The breakpoint junctions of these rearrangements often contain small overlapping sequences called “microhomology,” yet the genetic pathway(s) responsible have yet to be defined. We report a simple genetic system to detect microhomology-mediated repair (MHMR) events after a DNA double-strand break (DSB) in budding yeast cells. MHMR using >15 bp operates as a single-strand annealing variant, requiring the non-essential DNA polymerase subunit Pol32. MHMR is inhibited by sequence mismatches, but independent of extensive DNA synthesis like break-induced replication. However, MHMR using less than 14 bp is genetically distinct from that using longer microhomology and far less efficient for the repair of distant DSBs. MHMR catalyzes chromosomal translocation almost as efficiently as intra-chromosomal repair. The results suggest that the intrinsic annealing propensity between microhomology sequences efficiently leads to chromosomal rearrangements.

## Author Summary

Cancer results from an accumulation of mutations that transform a normal cell into one that proliferates uncontrollably. DNA double-strand breaks (DSBs) can lead to genetic mutations and chromosome rearrangements, underscoring the importance of functional DNA DSB repair pathways in the maintenance of chromosome integrity and tumor suppression. Ample evidence suggests that cells possess multiple DSB repair mechanisms with distinct mutational potentials, and one or more of these pathways is likely responsible for the formation of chromosomal translocations. Importantly, at the junctions of many rearrangements, small (2–20 bp in length) overlapping sequences from each of the original sequences, termed “microhomology,” are found, and they may provide a clue as to how these rearrangements form. Here, we describe our genetic investigation into how flanking microhomology influences the type and frequency of DSB repair. We also show that microhomology-mediated repair (MHMR) efficiently induces chromosomal translocations. This research provides a basic understanding of the mechanisms that utilize microhomology for mutagenic repair.

## Introduction

Chromosome structural variations such as deletions, duplications, inversions and chromosomal translocations contribute to evolution and genetic diseases [1], [2], [3]. A chromosomal translocation is a fusion between two non-homologous chromosomes. It contributes to cancer by forming a chimeric fusion protein or joining the regulatory region of one gene to the translated region of another gene, causing dysregulated gene expression [4], [5]. Altered expression of oncogenes or tumor suppressor genes then contributes to the development and progression of tumors [6]. Clinically, the detection of specific chromosomal translocations in patients can help in the diagnosis, treatment selection, and prognosis of the disease [4], [7], [8]. Substantial effort is therefore underway to characterize chromosomal translocation breakpoint junctions and the associated genetic changes. Puzzlingly however, much about how chromosomal translocations arise remains poorly understood. Such knowledge could constitute the first step to preventing their occurrence and curbing the chromosomal instability common in cancer cells.

DNA damage, in particular the DNA double strand break (DSB), is the inciting event in many chromosomal rearrangements [4], [5], [9], [10], [11]. Successful repair of DSBs avoids the persistence of toxic DNA lesions and maintains chromosomal integrity [12]. Accordingly, cells and organisms with a compromised DNA repair capacity demonstrate an elevated frequency of chromosomal translocations and chromosomal instability [5], [9]. Thus, all eukaryotic cells have two main pathways for repairing DSBs: non-homologous end-joining (NHEJ) and homologous recombination (HR). NHEJ joins two free DNA ends after a break by direct re-ligation whereas HR uses a homologous template for repair, most typically a sister chromatid [13], [14]. Nevertheless, neither canonical pathway is fully responsible for the formation of chromosomal translocations, as their repair products do not exhibit some of the key features of chromosomal translocation breakpoints described below [1], [15].

Recent technological advancement has allowed for the recovery and analysis of many breakpoint junctions from chromosomal rearrangements at the nucleotide level [2], [3], [16], [17]. These studies revealed that the breakpoint junctions often contain a few base pairs (2–20 bp) of overlapping sequences between joining chromosomal ends, and these small overlapping sequences are broadly called “microhomology” [18], [19]. The frequent presence of microhomology at breakpoints of chromosomal translocations could provide insight into the repair mechanisms used to form these chromosomal aberrations [16], [17], [18], [20]. Nevertheless, the precise mechanisms of microhomology-mediated DSB repair and its role in chromosomal translocations is not yet defined.

Recently, multiple microhomology-mediated repair (MHMR) pathways have been proposed to explain the usage of microhomology to repair DNA breaks. Microhomology-mediated end-joining (MMEJ) represents the Ku-independent end-joining repair process that anneals microhomologous sequences near the broken DNA ends [15], [21]. Synthesis-dependent-MMEJ (SD-MMEJ) creates *de novo* microhomology by transient templated synthesis at the DNA end [22]. Microhomology also facilitates distinct HR events such as microhomology-mediated synthesis-dependent strand annealing (MM-SDSA) that requires non-processive DNA synthesis and a *POL32*-independent “template switch” mechanism [23]. Microhomology-mediated break-induced replication (MM-BIR) operates as the RecA/Rad51-independent BIR to remove a collapsed replication fork, thereby producing the complex rearrangements found in copy number variations [1]. Microhomology/microsatellite-induced replication (MMIR) is genetically different from HR, NHEJ and MMEJ, producing segmental duplications from replication based DNA breakage [24]. These results suggest that the usage of microhomology is not a result of one defined repair pathway, but rather, the imprudent repair of breaks or collapsed replication forks relying on the intrinsic stability of annealed microhomology. Yet, the biological principles of microhomology-dependent repair, including how a particular pathway is selected among multiple repair options, are undefined.

Previously, we reported that MMEJ is particularly effective at repairing DSBs with non-complementary DNA ends. However, we realized that the previous assay system accidentally introduced a 12 bp imperfect microhomology sequence into the strain with non-complementary DNA ends but not to that with complementary ends, granting an unforeseen advantage [25]. These findings prompted a re-evaluation of the usage of microhomology flanking the DSB for repair. By systematically altering several key parameters, we determined the influence microhomology exerts on DNA DSB repair processes, and its contributions to the formation of reciprocal chromosomal translocations in a budding yeast model. We found that MHMR relies heavily on multiple factors that affect the stability of strand annealing between flanking microhomologous sequences. The stability of strand annealing dictates the efficiency of repair and the genetic factors, or pathway, involved. We also found that microhomology located on non-homologous chromosomes promotes chromosomal translocation. These results may provide the molecular basis for how DNA breaks lead to chromosomal rearrangements.

## Results

### Generation of a genetic system to detect MHMR events

**Figure 1:**
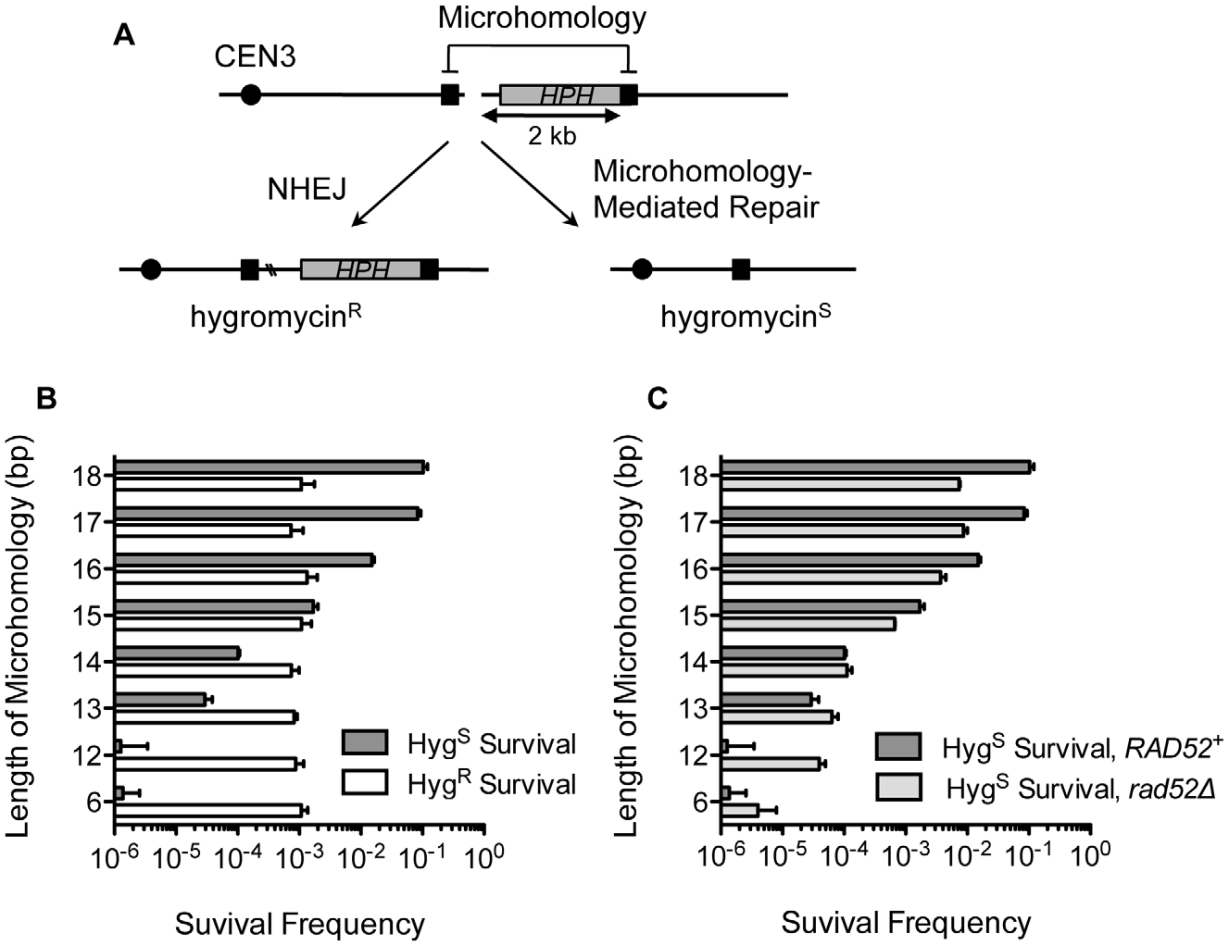
MHMR frequency increases 10-fold per nucleotide length from 12 bp to 17 bp. (A) A diagram of the genetic system used to distinguish microhomology-mediated repair from NHEJ-mediated repair. *HPH* represents the hygromycin B phosphotransferase gene that confers resistance to hygromycin treatment. The locations of HO cut site, microhomology, and centromere are shown. Two repair outcomes are distinguishable based on the sensitivity to hygromycin. Repair by NHEJ is shown by double crossing lines at the break site. (B) Graph showing survival frequency ± S.D. using the microhomology (Hyg^s^) and by NHEJ (Hyg^R^). Survival frequency was calculated by dividing the number of colonies surviving on the YEP-galactose plates by the number of colonies surviving on the YEPD plates. The results are the average of three independent experiments. (C) Survival frequency using the microhomology (Hyg^s^) is shown in wild type and *rad52*Δ. Survival frequency was calculated as shown in Figure 1B. The results are the average of three independent experiments ± S.D.

In order to study microhomology-mediated DSB repair, we devised a simple genetic system to efficiently score the frequency of MHMR events, distinguishing them from non-homologous end joining (NHEJ). Direct repeats of microhomology (6–18 bp) were placed on either side of an HO endonuclease recognition sequence at the *MAT* **a** locus, flanking the 2 kb hygromycin B phosphotransferase (*HPH*) gene that confers resistance to hygromycin B (Figure 1A). The microhomology sequence on the centromeric side of the DSB was only 2 bp from the HO cut-site. The strains lacked other HO recognition sites typically present in *HML* and *HMR,* and the HO gene was expressed under a galactose-inducible promoter (see Strains List in Table S1). Upon galactose-driven induction of HO, the recognition sequence was efficiently cleaved, creating a DNA DSB (data not shown). We then measured the survival frequency and the hygromycin resistance of the surviving colonies. If the break was repaired by NHEJ, the surviving colony was still hygromycin-resistant, but repair using the direct microhomology repeats led to hygromycin-sensitive survivors (Figure 1A). The types of repair events were further validated by recovering repair junctions by PCR and sequencing them (Table S2). Most (23 out of 24) hygromycin resistant events exhibited small base pair additions or deletions at the repair junction, typical of NHEJ events [26], whereas all hygromycin sensitive events resulted from a deletion of one of the repeats and the inter-repeat DNA, utilizing the microhomology for repair (Table S2). Deletion of *YKU70* or *DNL4* dramatically reduced hygromycin resistant survival, further validating the role of NHEJ in hygromycin resistant repair events (Table 1).

**Table 1:** Mutant strain survival frequencies.

	Microhomology-Mediated Survival			NHEJ-Mediated Survival		
Strain	Mean ± SD (×10^−2^)	bFold Change	cP Value	Mean ± SD (×10^−2^)	Fold Change	P Value
**YDV1.17** a	**8.3±1.0**	**1.00**	**–**	**0.073±0.042**	**1.00**	**–**
YDV1.17 *dnl4Δ*	6.6±2.2	0.79	0.40	<0.040^d^	<0.55	0.093
YDV1.17 *yku70Δ*	6.4±1.2	0.77	0.11	<0.030^d^	<0.41	0.093
YDV1.17 *rad52Δ*	0.87±0.14	0.10	0.0080	0.10±0.043	1.37	0.63
YDV1.17 *rad59Δ*	3.3±1.0	0.39	9.2×10^−6^	0.056±0.0057	0.76	0.57
YDV1.17 *rad52Δ rad59Δ*	0.0024±0.0031	0.0003	0.0051	0.27±0.044	3.71	0.0053
YDV1.17 *rad51Δ*	33.1±8.76	3.97	0.035	0.048±0.044	0.65	0.64
YDV1.17 *rad51Δ rad59Δ*	15.5±0.72	1.86	0.0030	0.058±0.050	0.79	0.68
YDV1.17 *rad52Δ rad59Δ rad51Δ*	0.88±0.097	0.11	0.0064	0.044±0.000018	0.59	0.34
YDV1.17 *exo1Δ sgs1Δ mre11-H125N*	0.88±0.24	0.11	0.0074	3.5±1.5	47.64	0.061
YDV1.17 *exo1Δ sgs1Δ*	0.69±0.17	0.08	0.0063	0.25±0.061	3.46	0.080
YDV1.17 *dnl4Δ rad52Δ*	1.2±0.16	0.15	0.0055	<0.0057^d^	<0.078	0.093
YDV1.17 *yku70Δ rad52Δ*	1.2±0.056	0.14	0.0073	0.0032±0.0056	0.04	0.12
YDV1.17 *pol32Δ*	0.67±0.21	0.08	0.0063	0.069±0.013	0.95	0.91
YDV1.17 *rev3Δ*	16.0±6.8	1.92	0.23	0.15±0.14	1.98	0.53
YDV1.17 *rad30Δ*	9.5±0.70	1.14	0.37	0.029±0.050	0.39	0.031
YDV1.17 *rev3Δ pol32Δ*	1.2±0.21	0.14	0.0076	0.069±0.023	0.94	0.79
YDV1.17 *rad30Δ pol32Δ*	1.3±0.30	0.16	0.0058	0.089±0.0034	1.21	0.57
YDV1.17 *rev3Δ rad30Δ*	17.0±2.5	2.04	0.041	0.044±0.077	0.60	0.60
YDV1.17 *rev3Δ rad30Δ pol32Δ*	1.3±0.040	0.15	0.0071	0.076±0.016	1.03	0.93
**YDV1.12** a	**0.00013±0.00022**	**1.00**	**–**	**0.087±0.031**	**1.00**	**–**
YDV1.12 *yku70Δ*	0.00078±0.00007	6.22	0.022	0.0022±0.0014	0.03	0.044
YDV1.12 *rad52Δ*	0.0039±0.0011	31.0	0.030	0.080±0.020	0.92	0.82
YDV1.12 *yku70Δ rad52Δ*	0.0042±0.0026	33.5	0.11	0.012±0.0046	0.14	0.059
YDV1.12 *rad59Δ*	0.00081±0.00023	6.46	0.0033	0.091±0.016	1.04	0.88
YDV1.12 *rad52Δ rad59Δ*	0.0020±0.00062	15.79	0.038	0.068±0.0093	0.78	0.50

SD, standard deviation; NHEJ, non-homologous end joining.

**^a^**Both YDV1.17 and YDV1.12 are compared to their mutant derivative strains, and not to each other. Thus, for both strains, the fold change is set to 1.00 and the p-value is not calculated, as indicated by “–” in the table.

b Fold change is calculated as the Mean _mutant_/Mean _wild type_.

c P-values are calculated using a two-tailed paired t-test by comparing mutant strains to the wild type parental strain.

d No hygromycin resistant colonies were detected in the triplicate experiments testing these mutants. Therefore, the hygromycin resistant survival was calculated as less than the average of 1 survivor in each of these experiments.

### The size of flanking microhomology affects the frequency of DSB repair

The successful development of a MHMR assay prompted us to characterize the necessary features of microhomology flanking a DSB that enable MHMR events. We first tested the effect of microhomology size on the DSB repair efficiency by changing the length of microhomology from 6 to 18 bps. As predicted, the length of microhomology exerted no effect on the NHEJ frequency; the hygromycin resistant (Hyg^R^) survival frequency was near constant (∼0.1%) for all yeast strains tested (Figure 1B). In contrast to NHEJ, the repair of the break using microhomology (and thus Hyg^S^ survival) increased as the length of microhomology increased (Figure 1B). The MHMR frequency of yeast with a DSB flanked by 17 bp microhomology corresponds to ∼10%, while those flanked by 6 or 12 bp is only ∼0.00001%. MHMR efficiency increased approximately 10-fold for every additional nucleotide of microhomology between 12 and 17 bp (Figure 1B).

Since the length of homology was a critical parameter for MHMR, we determined whether MHMR depends on the homology annealing factor Rad52, thus corresponding to a HR pathway variant. Surprisingly, deletion of *RAD52* led to distinctly different outcomes in MHMR frequency according to the length of microhomology. At the longer lengths of microhomology (15–18 bp), deletion of *RAD52* reduced MHMR frequency 3–10 fold, and a *rad52Δ rad59Δ* double gene deletion nearly abrogated all MHMR events (Figure 1C and Table 1). The *rad59Δ* alone also reduced microhomology-mediated repair, albeit more modestly than the *rad52Δ* (a 2.5-fold decrease versus a 10-fold decrease) (Table 1). However at shorter lengths (12–13 bps), Rad52 inhibited the usage of microhomology (Figure 1C). The results suggest that the size of microhomology is an important parameter for MHMR, and DSB repair using 15–18 bp microhomology falls under a Rad52-dependent repair mechanism.

### MHMR is more efficient when the microhomology is located close to the break

**Figure 2:**
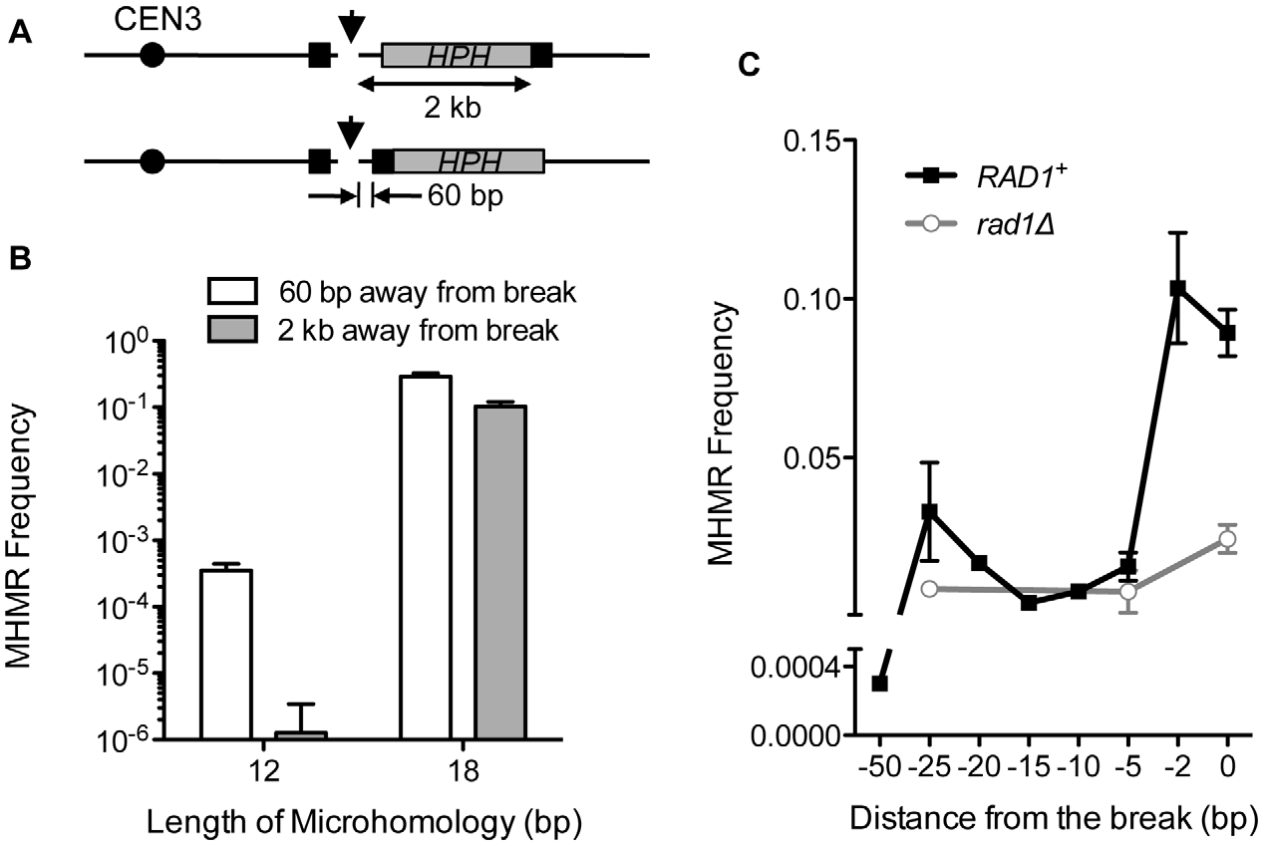
The distance from the DSB inversely affects MHMR frequency. (A) A diagram showing strains with microhomology inserted 2 kb and 60 bp from the DSB. *HPH* (gray box) represents the hygromycin B phosphotransferase gene. The position of the microhomology (black box) and the distance to the HO cleavage site are shown. (B) Survival using the microhomology for strains with 12 or 18 bp of microhomology located at 60 bp or 2 kb from the DSB. Survival frequency was calculated as shown in Figure 1B. For MHMR using microhomology at 60 bp from the DSB, the repair events were distinguished by sequencing of the repair junctions. The results are the average of three independent experiments ± S.D. (C) MHMR frequency using the 18 bp microhomology at various locations from the DSB in wild type and *rad1*Δ. Survival frequency was calculated as shown in Figure 1B. The results are the average of three independent experiments ± S.D.

To initiate the MHMR as seen in Figure 1C, we assumed that one or both microhomologies flanking a DSB should become single-stranded, and thus, DNA end resection is likely required for the repair process. Predictably, both *sgs1Δexo1Δ* and *sgs1Δexo1Δmre11-H125N*, that are deficient in end resection [27], [28], [29], demonstrated 10-fold decreases in the MHMR efficiency using the microhomology for repair (Table 1). Resection deficiency nevertheless increased NHEJ frequency dramatically, and thus, the total survival efficiency in these mutants was reduced only moderately (Table 1). We then positioned the telomere-proximal-side microhomology of 12 or 18 bp at two different locations from the break (60 bp or 2 kb) and tested whether the location of microhomology from the break affected MHMR frequency (Figure 2A). Since hygromycin resistance cannot discern the NHEJ events from the MHMR events in the strain carrying the microhomology located 60 bp from the break, we relied on sequencing to distinguish MHMR from NHEJ (Figure 2B). We found that the MHMR frequency of a DSB flanked by 18 bp of microhomology 60 bp apart was approximately 2.8-fold higher than 18 bp 2 kb apart, while the MHMR frequency of a DSB flanked by 12 bp of microhomology 60 bp apart was approximately 280-fold higher than 12 bp 2 kb apart (Figure 2B). These results suggest that the distance of the microhomology from the DNA break strongly influences the repair efficiency, especially when the size of microhomology is small.

### Microhomology-mediated repair requires 3′ flap removal

The DNA between the microhomology and the break constitutes a 3′ flap upon annealing of microhomology, and therefore, the location of the microhomology from the break dictates the 3′ flap size. Possibly, long non-homologous 3′ flaps destabilize annealing between microhomology and thereby reduce MHMR frequency. We thus examined whether the number and length of 3′ flaps affected MHMR by positioning one of the two microhomologies at several locations: immediately next to the DSB, or 5, 10, 15, 20, 25, and 50 bp away from the break, while the other microhomology on the telomere-proximal-side was fixed at 2 kb distal from the break. We found that a second flap size of 5 bp or longer strongly inhibited the MHMR process (Figure 2C). Deletion of *RAD1*, a single-strand DNA endonuclease that forms a complex with Rad10 and cleaves 3′ flap DNA [30], [31], reduced MHMR frequency 4-fold even in the strain having the single *HPH*-containing 3′ flap only (Figure 2C). MHMR in the absence of Rad1 was unaffected by the size of the 3′ flap next to the microhomology and occurred with similar efficiency in all of the strains. The results suggest that non-homologous 3′ flap removal is an important step of efficient MHMR and strongly influences survival frequency.

### Mismatched nucleotide sequence in microhomology inhibits MHMR

**Figure 3:**
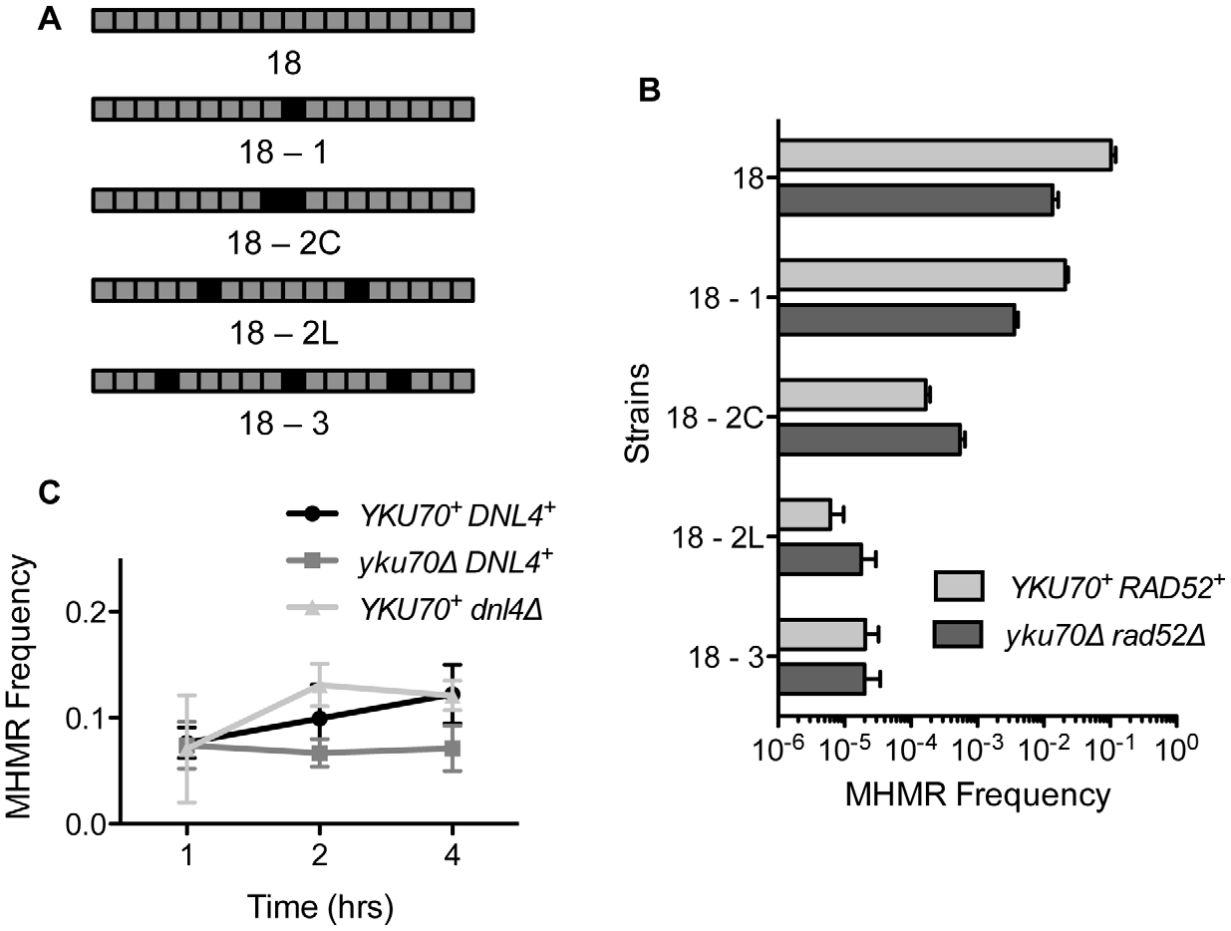
Mismatched microhomology inhibits repair efficiency, but NHEJ does not inhibit MHMR. (A) Illustration of strains with mismatched sequences. Black boxes indicate mismatched nucleotides. (B) MHMR frequency using 18 bp mismatched microhomology 2 kb from the DSB in wild type and *yku70*Δ *rad52*Δ. Survival frequency was calculated as shown in Figure 1B. The results are the average of three independent experiments ± S.D. (C) Graph showing MHMR frequency using the 17 bp of microhomology located 2 kb from the DSB in wild type, *yku70Δ* and *dnl4Δ* mutants. Survival frequency was calculated as shown in Figure 1B. The results are the average of three independent experiments ± S.D.

The presence of mismatches reduces the efficiency of the HR pathway [32], [33], [34], [35], [36], [37]. The presence of mismatches within the microhomology would also likely influence MHMR frequency by destabilizing the annealing process [31]. We examined whether mismatched sequences affect the frequency of MHMR events in our system. We replaced the 18 bp microhomology located 2 kb from the break with that carrying one, two, or three mismatched nucleotides at various positions and then measured the frequency of MHMR upon induction of HO endonuclease (Figure 3A). We found that mismatches effectively suppressed MHMR in both the wild type and the *yku70Δrad52Δ* mutant, but the inhibition was substantially greater in wild type. As the number of mismatches increased, the *yku70Δrad52Δ* mutant repaired the DNA break flanked by mismatched microhomology equally well, or even better than wild type, indicating that highly mismatched microhomology can be used to repair a DSB, albeit at a lower efficiency than perfectly matched homology (Figure 3B). The majority of the Hyg^S^ repair events in *yku70Δrad52Δ* mutants use the given mismatched microhomology, as confirmed by sequencing of the repair junctions. A few breakpoint junctions were not recovered, likely due to large deletions flanking the DSB using other endogenous microhomology similar to DSB repair events in *yku70Δ* mutants (Table S2) [25]. These results suggest that mismatches within the microhomology reduce MHMR efficiency.

### Neither NHEJ nor end configuration affects MHMR frequency

NHEJ has been shown to inhibit repair processes involving microhomology, both in yeast and mammals [21], [31], [38]. To test whether NHEJ also inhibits the MHMR process shown here, we expressed HO endonuclease for short intervals in the yeast strain carrying 17 bp of microhomology, and the MHMR frequency was compared in wild type, *yku70Δ* and *dnl4Δ* mutants, the last two of which are defective for NHEJ [39]. If NHEJ was inhibiting MHMR during pulsed induction, we expected an increase in the MHMR frequency in the NHEJ mutants. However, the wild type and both NHEJ mutants displayed similar frequencies of MHMR events (Figure 3C). The efficiency of repair was also similar to the repair during continuous HO endonuclease cleavage (compare Figure 1B and Figure 3C). We conclude that NHEJ does not inhibit MHMR in our experimental setting.

Previously, we reported that DSB repair by microhomology-mediated end joining occurred preferentially in a strain with two contemporaneous DNA breaks, which created broken DNA ends with no complementary base pairing potential [21], [25]. However, the previous assay system fortuitously introduced a 12 bp imperfect microhomology sequence in the strain with non-complementary DNA ends, facilitating MMEJ repair [21]. The efficient MHMR in our current studies, using a strain with a single HO cut-site and complementary ends, further challenges our earlier premise that MMEJ is restricted to specific end configurations. To resolve this issue, we constructed several yeast strains carrying one or two HO cut-sites, HO cut-sites from different *MAT* genes, and with complementary and non-complementary ends (Figure S1A). We found that microhomology could support DSB repair regardless of the end configuration of the induced DNA breaks (Figure S1B). These results broaden the utility of MHMR for the repair of different types of DSBs, regardless of end configuration.

### Genetics of MHMR

Mechanistically, microhomology could mediate DSB repair via mechanisms similar to break-induced replication (BIR) or single strand annealing (SSA). Rad51 is central to strand invasion for gene conversion and BIR, but inhibitory for SSA [40], [41], [42], [43], [44], [45]. In contrast, Rad59 is essential for SSA and for only a subset of gene conversion/BIR events [42], [45], [46], [47]. Therefore, the dependence of recombination events on Rad51 or Rad59 could offer mechanistic insights into the repair process. As shown in Table 1, we found that deletion of *RAD59* reduced recombination involving 17 bp microhomology nearly 3-fold. In contrast, deletion of *RAD51* improved MHMR frequency almost 4-fold. Deletion of both *RAD51* and *RAD59* rendered MHMR still more efficient than wild type. Deletion of *RAD51* also improved the MHMR frequency of *rad52Δ rad59Δ* to the level commensurate with that of *rad52Δ* mutant. The results suggest that MHMR may operate similarly to SSA albeit with unique redundancy between Rad52 and Rad59 [46].

We also examined the effect of *POL32* deletion on the MHMR frequency. Pol32 is an accessory protein for DNA polymerase δ, and it is dispensable for normal replication but essential for BIR and a subset of gene conversion pathways [48], [49], [50], [51]. Provided that MHMR is a SSA variant, we anticipated that Pol32 should be dispensable for MHMR. Surprisingly, deletion of *POL32* severely reduced MHMR frequency, decreasing it more than 12-fold (Table 1). The role of Pol32 in MHMR is not likely to recruit translesion polymerases because deletion of *REV3* and/or *RAD30* did not impact MHMR frequency (Table 1) [21]. The results suggest that MHMR is distinct from the established HR pathways as the genetic requirement is not consistent with either SSA or BIR. The inability to discern the type of repair events used for MHMR in our system by genetic tests prompted us to employ another assay listed below.

### MHMR does not require long-range break-induced replication

**Figure 4:**
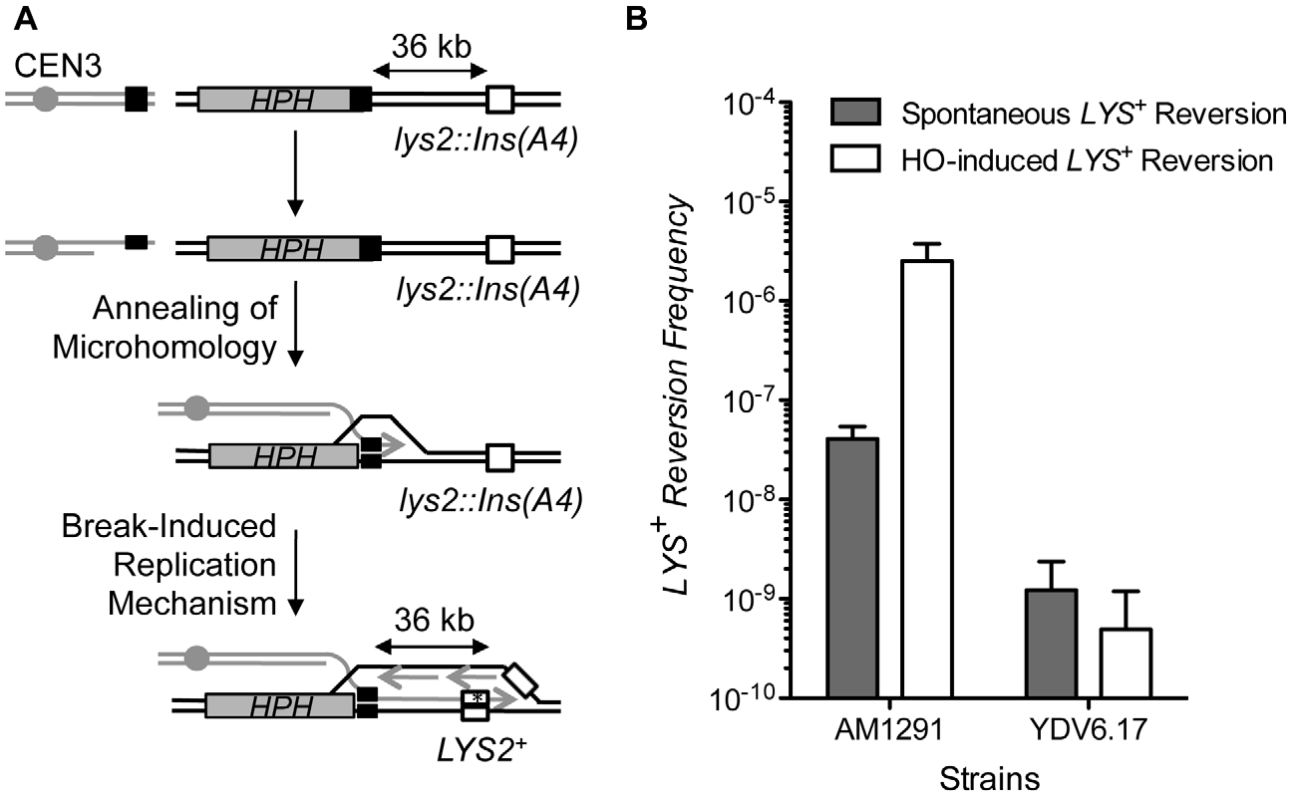
MHMR does not depend on long-range repair synthesis. (A) A diagram illustrating how microhomology repairs the break using a BIR mechanism. BIR has been shown to increase mutational frequency of *lys2::Ins(A4)* to *LYS2^+^* by frameshift mutation, which is shown as a white box [52]. The position of the centromere (gray circle), *HPH* (gray boxes) and the microhomology (black boxes) are shown. (B) Spontaneous and HO-induced *LYS^+^* reversion frequency was calculated using the average median value of five strains in three independent experiments in AM1291 that uses BIR to repair a DSB and *YDV6.17* that carries 17 bp of microhomology. The results are the average of the median values ± S.D.

In BIR, mutagenic DNA synthesis proceeds toward the end of the chromosome, yielding a high level of frameshift mutations at a *lys2::Ins(A4)* gene integrated 36 kb distal from the break [52]. In contrast, SSA likely does not involve repair synthesis at such a distant location and therefore should not lead to an increase in the *LYS2* frameshift mutation frequency (Figure 4A). Measuring the *LYS2* frameshift mutation frequency could thus help to discern if the repair mechanism involved SSA or BIR. We placed the *lys2::Ins(A4)* gene 36 kb from the HO cut-site flanked by 17 bp microhomology and measured the frequency of *LYS2* reversion (Figure 4A) [52]. As predicted, HO expression elevated (∼100 fold) the *LYS2* frameshift mutation frequency in the strain AM1291 that repaired a DSB by BIR [52]. However, the *LYS2* frameshift mutation did not increase after HO expression in the strain bearing microhomology flanking the break site (Figure 4B). The results suggest that MHMR operates differently than BIR, and likely resembles SSA.

### Pol32 is important for MHMR

Previous studies suggest that BIR and a subset of gene conversion repair processes are the only HR events dependent on Pol32 [50], [51]. We were puzzled that Pol32 played an important role in the MHMR events resembling SSA, and we further investigated why MHMR in our system relied heavily on Pol32. In our strain, the microhomology was located two nucleotides away from the HO cut, creating a very short (2 bp) non-homologous tail on the centromeric side of the DNA break. Such a short non-homologous tail could be removed by the proofreading activity of DNA polymerase δ [53]. We reasoned that Pol32 could catalyze MHMR by removing short 3′ flaps as part of the proofreading activity of polymerase δ. To test this idea, we measured the survival of *YDV1.18.0,* which bears a single long (2 kb long) telomeric 3′ flap but lacks a 2 bp 3′ flap on the centromeric side in the *pol32* mutant (Figure S2A). We found that microhomology-dependent repair in *YDV1.18.0* was still dependent on Pol32, suggesting that Pol32 is required for step(s) other than short 3′ flap removal (Figure S2B). Alternatively, we hypothesized that Pol32 may be involved in the repair synthesis from the annealed microhomology, further stabilizing the interaction of the annealed DNA duplex. In this model, the shorter the length of homology, the more important Pol32 would be for repair. We examined the survival frequency of the *pol32*-deleted strains carrying 205 bp (*EAY1141*) or 1.3-kb (*YMV80*) direct repeat sequences (Figure S2A and S2B). We found that deletion of *POL32* decreased survival frequency moderately (0.64-fold) in EAY1141 but not at all in YMV80 (Figure S2B). These results suggest that Pol32 is important for recombination using short stretches of homology.

### MHMR leads to chromosomal translocations

**Figure 5:**
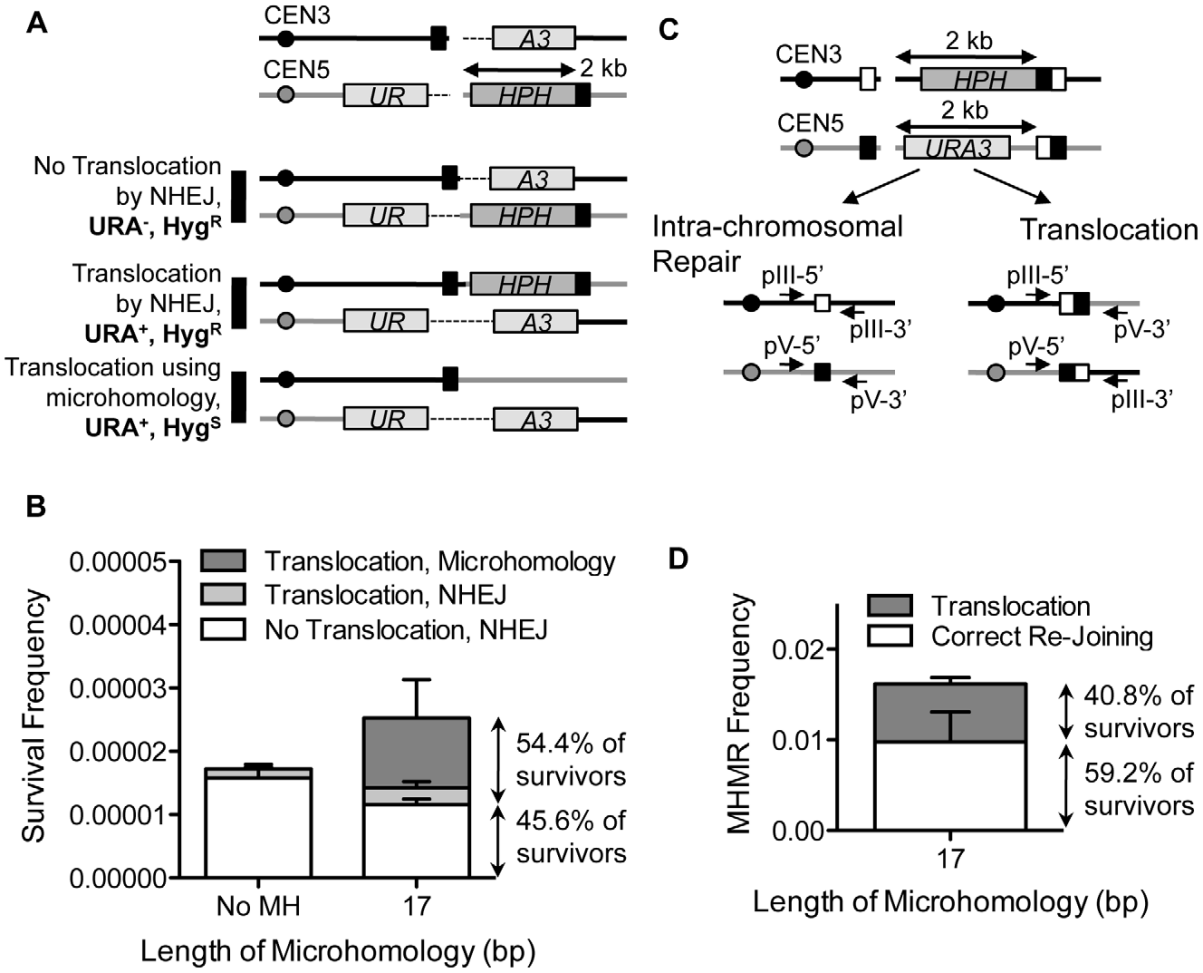
MHMR stimulates HO break-induced chromosomal translocations. (A) Diagram of the strain with one HO recognition sequence on chromosome III and one HO recognition sequence on chromosome V. 17 bp of microhomology (shown in black box) found on the centromeric side of the DSB on chromosome III is also found on the telomeric side of the DSB on chromosome V, 2 kb from the break site. In this strain, one part of the *URA3* gene (*UR*) is found on the centromeric side of chromosome V, and the other part of the *URA3* gene (*A3*) is found on the telomeric side of chromosome III. Since artificial introns (the dotted lines) are inserted between the gene and the HO cut-site, the *URA3* gene is expressed in the event of reciprocal translocation. Three possible repair outcomes are shown at the bottom. (B) The frequencies of the repair outcomes from the yeast strain with breaks on two different chromosomes are shown. Survival frequency was calculated as shown in Figure 1B. The results are the average of three independent experiments ± S.D. (C) Diagram illustrating the strain that allows for competition between intra-chromosomal and inter-chromosomal MHMR. The positions of microhomology (white and black boxes), and *HPH* and *URA3* markers are shown. The location of sequences homologous to two sets of primers used to check the types of repair events are shown in arrows. (D) Survival frequency of intra- and inter-chromosomal MHMR. Survival frequency was calculated as shown in Figure 1B. The results are the average of three independent experiments ± S.D. 100 colonies from each survival experiment were assessed by PCR to detect intra-chromosomal or inter-chromosomal repair products.

The frequent presence of microhomology at breakpoint junctions in chromosomal rearrangements prompted us to test whether MHMR could promote chromosomal translocations [16], [17], [18], [20]. To measure the frequency of MHMR between two non-homologous chromosomes, we placed the 17 bp microhomology sequence on the centromeric side of chromosome III and the telomeric side of chromosome V, flanking HO cut-sites on both chromosomes (Figure 5A). We expected three ways to repair these two breaks: (1) NHEJ without translocation, (2) chromosomal translocation by NHEJ, and (3) chromosomal translocation by MHMR of one junction and NHEJ of the other junction (Figure 5A). With continuous induction of HO endonuclease, the incidence of chromosomal translocation increased from an average of 10.7% of survivors suffering an NHEJ-mediated translocation to an additional 43.7% of survivors suffering a microhomology-mediated translocation, bringing to total translocation frequency to 54.4% of all survivors. Therefore, the presence of the microhomology on the non-homologous chromosome increased the total frequency of chromosomal translocation (Figure 5B).

In SSA, inter-chromosomal repair occurs as efficiently as intra-chromosomal repair [54]. We thus tested whether MHMR could catalyze chromosomal translocations as efficiently as intra-chromosomal events. To test this idea, we constructed a yeast strain that could repair both breaks by MHMR intra-chromosomally and inter-chromosomally (Figure 5C). 99.3% of survivors lost both *HPH* and *URA3* marker genes and repaired the breaks by MHMR. Importantly, of those survivors, 40.8% survived by reciprocal chromosomal translocation (Figure 5D). The MHMR-mediated chromosomal translocation was dependent on end resection because deletion of *SGS1 EXO1* severely reduced the frequency of chromosomal translocation (Table S3). The results indicate that intra- and inter-chromosomal MHMR occur with almost equal frequency, similar to SSA. These results may partially explain why microhomology is so often found at the breakpoint junctions of chromosomal rearrangements.

## Discussion

By inserting various sizes of microhomology with or without mismatches at multiple locations flanking a DSB, we systematically addressed the role of microhomology in the repair of a DNA break and the formation of chromosomal translocations. The results demonstrate that more than one mechanism exists for catalyzing microhomology-mediated repair. Involvement of different mechanisms, carried out by different genetic factors, depends on the location and the length of microhomology. We also demonstrated that MHMR lacks preference for intra-chromosomal repair and promotes high levels of chromosomal translocations. Our results uncovered the surprising complexity of MHMR processes and mutagenic potential.

Successful MHMR depends on the size and location of the microhomology and the presence of mismatches, because these parameters strongly affect the frequency and type of MHMR (Figure 1, Figure 2, and Figure 3). The genetic requirements for MHMR are also radically different between those for shorter or longer microhomology; repair of a DSB using microhomology 15 bp and longer is Rad52 dependent whereas repair involving less than 15 bp microhomology is inhibited by Ku and Rad52 (Table 1). Furthermore, the repair events mediated by 17 or 18 bp of microhomology do not fully conform to the genetic requirements for typical SSA, as they become heavily dependent on Pol32 and either Rad52 or Rad59. MHMR thus resembles MMIR that repairs broken replication forks and produces Pol32-dependent segmental duplications [24].

These results, along with evidence from several other studies [1], [15], [22], [50] suggest that microhomology directs multiple different repair events, many of which are the variants of established repair mechanisms but with distinct genetic and thus, mechanistic differences. All of these pathways may exploit the stability of annealed microhomology to strengthen the association between broken DNA ends. The size, the degree of homology, and the location with respect to the break all contribute to the thermodynamic energy of strand annealing and dictate the repair mechanism and the repair outcomes, utilizing the biochemical activities of various DNA repair enzymes (Table S4) [15], [21]. Thus, the most distinguishing feature of this process may be the genetic and mechanistic complexity of MHMR, and we speculate that this complexity may have evolved to deal with a wide range of DNA lesions induced by toxic chemicals and metabolites. However, this adaptability poses a significant challenge for the establishment of its genetic attributes, and similar conclusions were proposed to account for the genetic plasticity of NHEJ [55].

Additionally, these results raise a concern about the validity of some of the earlier results pertaining to MHMR, because these studies did not consider the possibility that MHMR may consist of multiple pathways encompassing widely different genetic and mechanistic requirements. Realization of this complexity challenges the generalization of all repair events that occur in the absence of a certain gene and use various lengths of microhomology as mechanistically common MHMR events.

**Figure 6:**
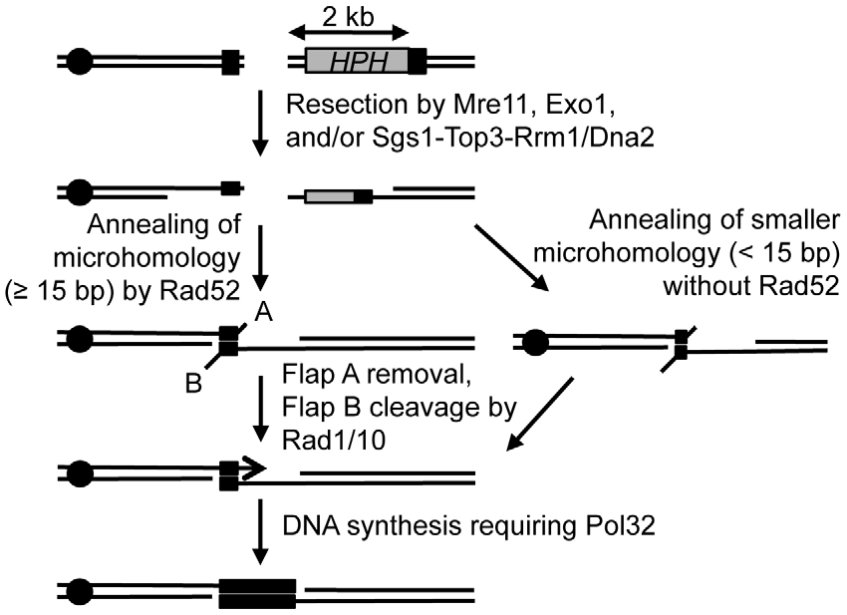
Proposed mechanism for repair of a DSB using microhomology. After a break, ends are resected to single-stranded DNA, and microhomology flanking the break (shown in black boxes) are brought together and annealed. Rad52 facilitates repair using microhomology that is 15 bp or longer, and microhomology less than 15 bp is inhibited by Rad52. A 2 bp 3′ flap (A) is removed, likely through the proof-reading activity of Polymerase δ, and a 2 kb 3′ flap (B) is removed by the Rad1/Rad10 heterodimer endonuclease. Pol32 stabilizes the annealed microhomology and the break is healed, deleting the intervening DNA and one of the microhomology sequences, similar to SSA. The location of the centromere (black circle) is shown.

Despite the unique challenges associated with analyzing MHMR events, we demonstrated that MHMR using longer than 15 bp of microhomology in our experimental system operates as the SSA but not the BIR variant. Our conclusion is based on the fact that the mutagenic BIR mechanism would need to replicate the DNA all the way to the end of the chromosome [52], and our data indicated that there was no such low-fidelity replication occurring 36 kb from the break-site in the *lys2::Ins(A4)* fluctuation assay. According to the sum of the functional assays and mutant analysis, we have compiled a model of the microhomology-mediated pathway (Figure 6). The model proposes that resection induces the onset of MHMR, allowing for the single-stranded microhomology to anneal. Rad52 facilitates annealing between microhomology if the microhomology length is 15 bp or longer. However, if the microhomology length is shorter than 15 bp, Rad52 prevents annealing of microhomology and thereby inhibits mutagenic deletions in the DNA. This inhibition of MHMR between short lengths of microhomology may be attributed to the minimum size of ssDNA catalyzed by Rad52 for annealing [56], yet no such information is available. We also found that Rad59 contributes to MHMR especially when Rad52 is absent. Surprisingly, the deletion of *RAD51* offset the role of Rad59 in MHMR, bringing survival frequency of the *rad52 rad59 rad51* triple mutant to the equivalent of a single *rad52* deletion (Table 1). Rad59 could thus facilitate MHMR by neutralizing the inhibitory activity of Rad51, as has been previously reported [57].

Upon successful annealing between microhomology, Rad1/Rad10 cleaves 3′ flap DNA, and Pol32 stabilizes the annealing intermediate between single-strand DNA to allow for Polδ to extend the annealed homologous sequence and complete the repair process (Figure 6). We suggest that Pol32 likely functions similarly for BIR and a subset of gene conversion events to maximize the stability of strand pairing. Previous work studying the SSA pathway found that repeats of 29 bp were used 0.2% of the time to repair a DSB [46]. Yet, our work found that 17 bp repeats efficiently repair the DSB with ∼10% survival efficiency. We hypothesized that this high efficiency of repair was due to the microhomology location at the very end of the DNA, and that 3′ flap removal may hinder this repair process. Accordingly, the DNA sequence at the end of the broken DNA is much more catalytic for microhomology-mediated deletions and translocations, as a 5 bp nucleotide flap inhibits repair 10-fold (Figure 2C). We speculate that the instability of the annealed intermediate in MHMR can be offset by the lack of a second tail or by the extension of annealed sequences via repair synthesis initiated from DNA end without further processing. Our results also showed that MHMR can process a second flap independently of Rad1, further emphasizing the difference between SSA and microhomology-mediated SSA. The presence of a Rad1-independent mechanism to remove 3′ flaps has been proposed before but the genetics and the mechanisms of such pathway(s) are not identified yet. We propose that such pathway(s) can efficiently catalyze MHMR with two flaps in the absence of Rad1/10 endonuclease.

Most importantly, we showed that the presence of microhomology across the break between different chromosomes dramatically promotes the formation of chromosomal translocations (Figure 5). The results may explain why women with an increased familial risk of breast cancer and breast cancer patients themselves have a higher frequency of MHMR and SSA repair pathways in their white blood cells [58]. The increased frequency of microhomology-mediated chromosomal translocations is partly due to the lack of bias to intra-chromosomal repair as seen in the repair events by SSA. The results are the direct opposite of NHEJ, which shows a strong bias to intra-chromosomal repair and suppression of chromosomal translocations [59]. Evidence has emerged that ATM-dependent end-tethering suppresses inter-chromosomal end joining and thereby suppresses break-induced chromosomal translocations [59], [60]. We propose that either end-tethering does not inhibit inter-chromosomal SSA or becomes nonfunctional at the time of the SSA or MHMR process. Regardless, these results shed light on how MHMR induces chromosomal translocations.

Evidence indicates that breakpoint junctions of chromosomal rearrangements in humans contain microhomology of 2–20 bp and such events are markedly elevated in NHEJ deficient cells [2], [15]. The findings that MHMR efficiently catalyzes chromosomal translocations support its contributions to chromosomal translocation formation in humans. However, most breakpoint junctions of chromosomal translocations in human studies show 1–6 bp microhomology, which is much shorter than that used for MHMR events described in this study. To account for this discrepancy, we surmise that the usage of microhomology is dictated not only by the efficiency of such sequence to catalyze MHMR but also the frequency of available flanking microhomology. The low efficiency of MHMR using shorter microhomology can be offset by the high availability of shorter microhomology and thus their frequent appearance at breakpoints in chromosomal aberrations in humans. Additional difference in end processing and/or repair protein activity such as Rad52 or resection enzyme(s) among species may disproportionally favor the usage of certain size microhomology in MHMR. Further study into understanding MHMR pathways and their regulation could lead to the etiology of chromosomal aberrations in patients with a higher baseline of mutagenic DNA repair processes.

## Methods

### Strains

All yeast strains are derived from JKM139 or JKM179 [26], [61]. The genotype of JKM139 is *hoΔ MAT* ***a*** *hmlΔ::ADE1 hmrΔ::ADE1 ade1-100 leu2-3,112 lys5 trp1::hisG ura3-52 ade3::GAL-HO.* The genotype for JKM179 is *hoΔ MATα hmlΔ::ADE1 hmrΔ::ADE1 ade1-100 leu2-3,112 lys5 trp1::hisG ura3-52 ade3::GAL-HO* (Table S1). YDV strains and their derivatives were made by amplification of the *HPH* gene from pAG26 with 90-bp oligonucleotides, containing 20-bp of homology to *HPH*, various sizes of microhomology sequence, and homology to the Z1 region of *MAT* ***a*** on chromosome III. Gene deletion mutants were constructed by PCR-based one step gene deletion technique using oligonucleotides flanked by terminal sequences homologous to the open reading frame of target genes [62].

### HO endonuclease induction

Logarithmically growing yeast cells were incubated in YEP-Glycerol for 16 hours, and serial dilutions were plated onto YEPD and YEP-galactose plates. Galactose induces HO endonuclease expression [21]. To induce HO expression for shorter duration, 2% (w/v) galactose was added to logarithmically growing yeast cells in YEP-glycerol medium, and after the indicated time of incubation, aliquots of culture were removed and plated onto YEPD to inhibit further HO endonuclease expression [59]. Survival frequency was calculated by dividing the number of colonies surviving on YEP-galactose by the number of colonies surviving on YEPD plate. The plates were replica-plated on hygromycin-containing or uracil-deficient plates to determine whether they retained the *HPH* or *URA3* genes, respectively.

### BIR test

Logarithmically growing yeast cells in YEP-glycerol media were harvested by centrifugation and re-suspended to a concentration of 5×10^8^ cells/ml. Cells (2×10^8^ cells) were plated on 150 mm lysine drop-out plates containing dextrose or galactose. The median of five strains was taken for each experiment. Each experiment was repeated three times with five strains each, and an average median value was calculated. Spontaneous *LYS^+^* reversion frequency was calculated from the number of colonies on the lysine^−^ plates, and BIR-induced *LYS^+^* reversion frequency was calculated from the number of colonies on the lysine^−^ galactose-containing plates [52]. The entire experiment (five single colonies per strain) was repeated two more times for triplicate values.

### Statistical analysis

Unless otherwise stated, all experiments were conducted in triplicate, so that an average and standard deviation were calculated. P-values were calculated for mutants as compared to the respective wild type strain using a two-tailed paired t-test.

## Supporting Information

Figure S1 MHMR occurs independently of end configuration or number of DSBs. (A) To test if microhomology-mediated repair operates regardless of yeast mating type or the number of DSBs, several strains were constructed with 13 or 17 bp of microhomology (only 17 bp is shown) bearing a different mating type (YDV100), or number of HO cleavage sites producing complementary (YDV200) or non-complementary (YDV300) overhangs. The centromere (black circle) is shown, along with the *URA3* and *HPH* marker genes (gray boxes), and the *MATa* microhomology (black box) and the *MATα* microhomology (white box). (B) Survival frequency was calculated as shown in Figure 1B. The results are the average of three independent experiments ± S.D.
(PDF)

Figure S2 Pol32 is important for stabilizing microhomology for repair, not for 3′ flap removal. (A) Diagram of strains with 18 bp, 205 bp or 1.3 kb of homologous repeats, also with various 3′ flap lengths. (B) Table demonstrating that Pol32 becomes more important as the length of homology decreases. Survival frequency was calculated as shown in Figure 1B. The results are the average of three independent experiments. The p-value is calculated using a two-tailed paired t-test. Both the fold change and the p-value compare each mutant strain to the respective wild type parental strain.
(PDF)

Table S1 Strains list.
(DOC)

Table S2 Sequencing data.
(DOC)

Table S3 MHMR chromosomal translocations require resection.
(DOC)

Table S4 Melting temperatures.
(DOC)
