# Supplementary material for: Microhomology Directs Diverse DNA Break Repair Pathways and Chromosomal Translocations
Source: PLoS Genet. 2012 Nov 8;8(11):e1003026. doi: 10.1371/journal.pgen.1003026 (PMC3493447; doi:10.1371/journal.pgen.1003026)
Supplement: Figure S1 — MHMR occurs independently of end configuration or number of DSBs. (A) To test if microhomology-mediated repair operates regardless of yeast mating type or the number of DSBs, several strains were constructed with 13 or 17 bp of microhomology (only 17 bp is shown) bearing a different mating type (YDV100), or number of HO cleavage sites producing complementary (YDV200) or non-complementary (YDV300) overhangs. The centromere (black circle) is shown, along with the URA3 and HPH marker genes (gray boxes), and the MATa microhomology (black box) and the MATα microhomology (white box). (B) Survival frequency was calculated as shown in Figure 1B. The results are the average of three independent experiments ± S.D. (PDF) [file pgen.1003026.s001.pdf]

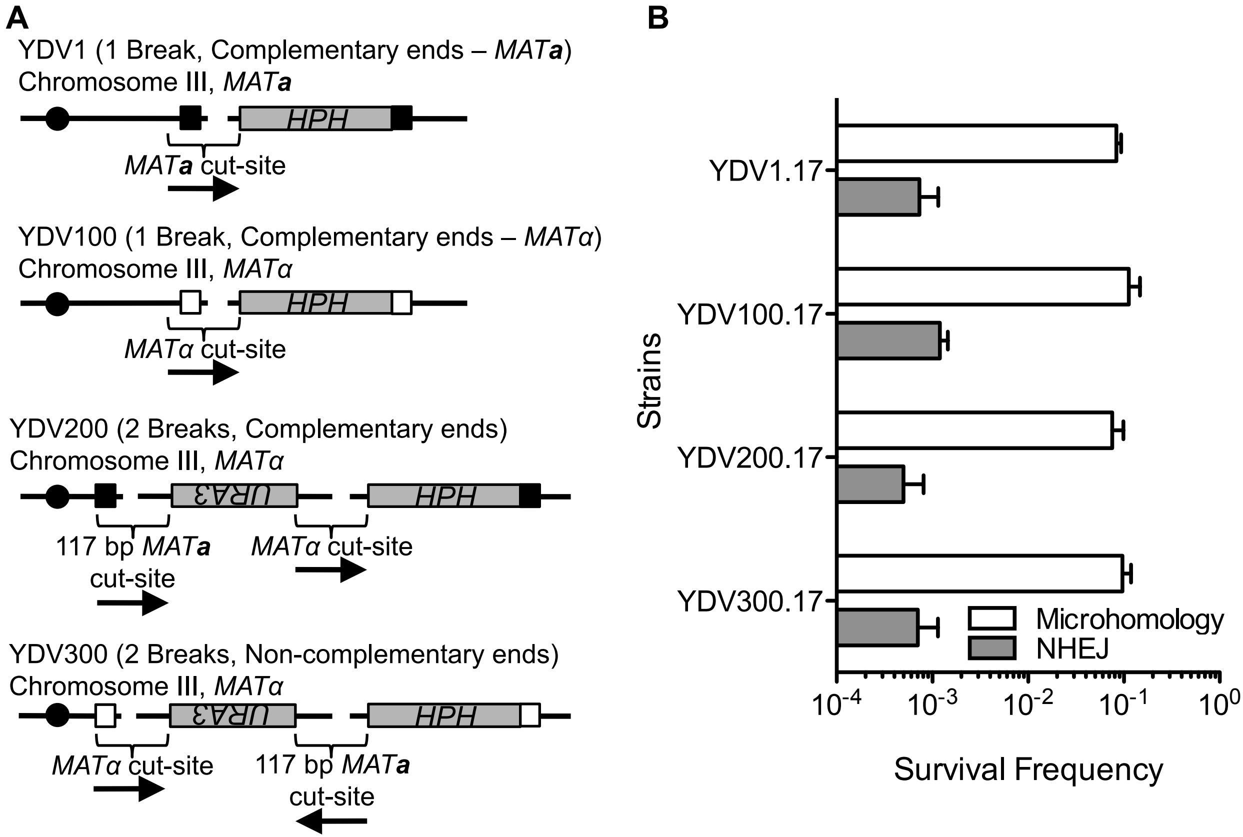
A
YDV1 (1 Break, Complementary ends – MATa)
Chromosome III, MATa
HPH
MATa cut-site
YDV100 (1 Break, Complementary ends – MATα)
Chromosome III, MATα
HPH
MATα cut-site
YDV200 (2 Breaks, Complementary ends)
Chromosome III, MATα
URA3
HPH
117 bp MATa cut-site
MATα cut-site
YDV300 (2 Breaks, Non-complementary ends)
Chromosome III, MATα
URA3
HPH
MATα cut-site
117 bp MATa cut-site
B
Strains
YDV1.17
YDV100.17
YDV200.17
YDV300.17
Microhomology
NHEJ
10-4
10-3
10-2
10-1
100
Survival Frequency
