# Supplementary material for: Microhomology Directs Diverse DNA Break Repair Pathways and Chromosomal Translocations
Source: PLoS Genet. 2012 Nov 8;8(11):e1003026. doi: 10.1371/journal.pgen.1003026 (PMC3493447; doi:10.1371/journal.pgen.1003026)
Supplement: Figure S2 — Pol32 is important for stabilizing microhomology for repair, not for 3′ flap removal. (A) Diagram of strains with 18 bp, 205 bp or 1.3 kb of homologous repeats, also with various 3′ flap lengths. (B) Table demonstrating that Pol32 becomes more important as the length of homology decreases. Survival frequency was calculated as shown in Figure 1B. The results are the average of three independent experiments. The p-value is calculated using a two-tailed paired t-test. Both the fold change and the p-value compare each mutant strain to the respective wild type parental strain. (PDF) [file pgen.1003026.s002.pdf]

A

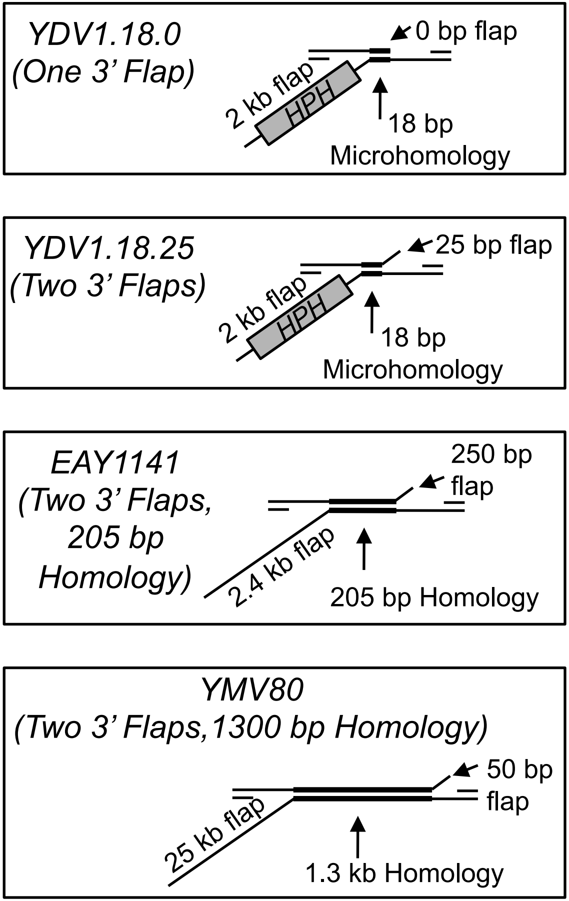

B

Strain	***Hyg^S Surv.***	***Std. Dev.***	***Fold-Change***	***P-Value***
YDV1.18.0	0.089	0.0073	1.00	--
YDV1.18.0 pol32Δ	0.011	0.0026	**0.12**	**0.0026**
YDV1.18.25	0.033	0.0155	1.00	--
YDV1.18.25 pol32Δ	0.0010	0.00020	**0.03**	**0.00020**

Strain	***Hyg^S Surv.***	***Std. Dev.***	***Fold-Change***	***P-Value***
EAY1141	0.97	0.172	1.00	--
EAY1141 pol32Δ	0.62	0.048	**0.64**	**0.071**
YMV80	0.75	0.044	1.00	--
YMV80 pol32Δ	0.79	0.021	**1.05**	**0.394**
