# Supplementary material for: Microhomology Directs Diverse DNA Break Repair Pathways and Chromosomal Translocations
Source: PLoS Genet. 2012 Nov 8;8(11):e1003026. doi: 10.1371/journal.pgen.1003026 (PMC3493447; doi:10.1371/journal.pgen.1003026)
Supplement: Table S1 — Strains list. (DOC) [file pgen.1003026.s003.doc]

**Table S**1. Strains List.

| **Strain** | **Genotype** | **Reference** |
| --- | --- | --- |
| JKM139 | *hoΔ MAT****a*** *hmlΔ::ADE1 hmrΔ::ADE1 ade1-100 leu2-3,112 lys5 trp1::hisG ura3-52 ade3::GAL-HO* |  |
| JKM179 | *hoΔ MATα hmlΔ::ADE1 hmrΔ::ADE1 ade1-100 leu2-3,112 lys5 trp1::hisG ura3-52 ade3::GAL-HO* | [[1]](#_ENREF_1) |
| SLY18 | *ho∆ MATα::URA3::HOcs hml∆::ADE1 hmr∆::ADE1 ade1-100 leu2-3,112 lys5 trp1::hisG ura3-52 ade3::GAL::HO* |  |
| SLY60 | *ho∆ MAT∆3’::intron::ura3∆5’ hml∆::ADE1 hmr∆::ADE1 ade1-100 leu2-3,112 lys5 trp1::hisG ura3∆3’::intron::HOcs ade3::GAL::HO (thus his-)* |  |
| YZZ115 | JKM139 *sgs1*∆*::KAN* *exo1*∆*::TRP1* |  |
| YZZ535 | JKM139 *sgs1*∆*::KAN* *exo1*∆*::TRP1 mre11-H125N::URA3* |  |
| AM1291 | *MAT****a****-LEU2-tel/MAT****a****-inc ade1 met13 ura3-52 leu2-3,112/leu2-3,112 thr4 hmlΔ::ADE1/hmlΔ::ADE3 hmrΔ::HYG ade3::GAL-HO FS2D::NAT lys2::Ins(A4) on Chr III at the “16-kb” position* |  |
| EAY1141 | *hoΔ mat::leu2::hisG hmrΔ3 thr4 leu2 trp1 THR4-ura3-A(205bp)-HOcs-URA3-A ade3::GAL10-HO::NatMX* |  |
| YMV80 | *hoΔ hml∆::ADE1 MATα∆::hisG hmr∆::ADE1 leu2-cs ade3::GAL::HO ade1 lys5 ura3-5, leu2::CS* |  |
| YDV1.18 | *JKM139 MAT****a****Z1::HPH::18bp microhomology* | This Study |
| YDV1.17 | *JKM139 MAT****a****Z1::HPH::17bp microhomology* | This Study |
| YDV1.16 | *JKM139 MAT****a****Z1::HPH::16bp microhomology* | This Study |
| YDV1.15 | *JKM139 MAT****a****Z1::HPH::15bp microhomology* | This Study |
| YDV1.14 | *JKM139 MAT****a****Z1::HPH::14bp microhomology* | This Study |
| YDV1.13 | *JKM139 MAT****a****Z1::HPH::13bp microhomology* | This Study |
| YDV1.12 | *JKM139 MAT****a****Z1::HPH::12bp microhomology* | This Study |
| YDV1.6 | *JKM139 MAT****a****Z1::HPH::6bp microhomology* | This Study |
| YDV2.18 | *YDV1.18 rad52Δ::KAN* | This Study |
| YDV2.17 | *YDV1.17 rad52Δ::KAN* | This Study |
| YDV2.16 | *YDV1.16 rad52Δ::KAN* | This Study |
| YDV2.15 | *YDV1.15 rad52Δ::KAN* | This Study |
| YDV2.14 | *YDV1.14 rad52Δ::KAN* | This Study |
| YDV2.13 | *YDV1.13 rad52Δ::KAN* | This Study |
| YDV2.12 | *YDV1.12 rad52Δ::KAN* | This Study |
| YDV2.6 | *YDV1.6 rad52Δ::KAN* | This Study |
| YDV3.18 | *YDV1.18 yku70Δ::KAN* | This Study |
| YDV3.17 | *YDV1.17 yku70Δ::KAN* | This Study |
| YDV3.12 | *YDV1.12 yku70Δ::KAN* | This Study |
| YDV3.6 | *YDV1.6 yku70Δ::KAN* | This Study |
| YDV4.18 | *YDV1.18 yku70Δ::KAN rad52Δ::TRP* | This Study |
| YDV4.17 | *YDV1.17 yku70Δ::KAN rad52Δ::TRP* | This Study |
| YDV4.12 | *YDV1.12 yku70Δ::KAN rad52Δ::TRP* | This Study |
| YDV1.18.0 | *JKM139 MAT****a****Z1::HPH::18bp MH, 0bp flap* | This Study |
| YDV1.18.5 | *JKM139 MAT****a****Z1::HPH::18bp MH, 5bp flap* | This Study |
| YDV1.18.10 | *JKM139 MAT****a****Z1::HPH::18bp MH, 10bp flap* | This Study |
| YDV1.18.15 | *JKM139 MAT****a****Z1::HPH::18bp MH, 15bp flap* | This Study |
| YDV1.18.20 | *JKM139 MAT****a****Z1::HPH::18bp MH, 20bp flap* | This Study |
| YDV1.18.25 | *JKM139 MAT****a****Z1::HPH::18bp MH, 25bp flap* | This Study |
| YDV1.18.50 | *JKM139 MAT****a****Z1::HPH::18bp MH, 50bp flap* | This Study |
| YDV5.18.0 | *YDV1.18.0 rad1Δ::KAN* | This Study |
| YDV5.18.5 | *YDV1.18.5 rad1Δ::KAN* | This Study |
| YDV5.18.25 | *YDV1.18.25 rad1Δ::KAN* | This Study |
| YDV6.17 | *JKM139 MAT****a****Z1::HPH::17bp MH lys2::Ins(A4) on Chr III at the “36-kb” position* | This Study |
| YDV1.18.1MS | *JKM139 MAT****a****Z1::HPH::18bp with 1 central mismatch* | This Study |
| YDV1.18.2CMS | *JKM139 MAT****a****Z1::HPH::18bp with 2 central mismatches* | This Study |
| YDV1.18.2LMS | *JKM139 MAT****a****Z1::HPH::18bp with 2 lateral mismatches* | This Study |
| YDV1.18.3MS | *JKM139 MAT****a****Z1::HPH::18bp with 3 mismatches* | This Study |
| YDV3.18.1MS | *YDV1.18.1MS yku70Δ::KAN* | This Study |
| YDV3.18.2CMS | *YDV1.18.2CMS yku70Δ::KAN* | This Study |
| YDV3.18.2LMS | *YDV1.18.2LMS yku70Δ::KAN* | This Study |
| YDV3.18.3MS | *YDV1.18.3MS yku70Δ::KAN* | This Study |
| YDV4.18.1MS | *YDV1.18.1MS yku70Δ::KAN rad52Δ::TRP* | This Study |
| YDV4.18.2CMS | *YDV1.18.2CMS yku70Δ::KAN rad52Δ::TRP* | This Study |
| YDV4.18.2LMS | *YDV1.18.2LMS yku70Δ::KAN rad52Δ::TRP* | This Study |
| YDV4.18.3MS | *YDV1.18.3MS yku70Δ::KAN rad52Δ::TRP* | This Study |
| YDV7.17 | *YDV1.17 pol32Δ::KAN* | This Study |
| YDV7.18.0 | *YDV1.18.0 pol32Δ::KAN* | This Study |
| YDV7.18.25 | *YDV1.18.25 pol32Δ::KAN* | This Study |
| YDV7.205 | *EAY1141 pol32Δ::KAN* | This Study |
| YDV7.1300 | *YMV80 pol32Δ::KAN* | This Study |
| YDV8.17 | *YDV1.17 dnl4Δ::KAN* | This Study |
| YDV9.17 | *YDV1.17 rad59Δ::TRP* | This Study |
| YDV9.12 | *YDV1.12 rad59Δ::TRP* | This Study |
| YDV10.17 | *YDV1.17 rad51Δ::KAN* | This Study |
| YDV12.17 | *YDV1.17 rad51Δ::KAN rad59Δ::TRP* | This Study |
| YDV13.17 | *YDV1.17 rad52Δ::KAN rad59Δ::TRP* | This Study |
| YDV13.12 | *YDV1.12 rad52Δ::KAN rad59Δ::TRP* | This Study |
| YDV14.17 | *YDV1.17 dnl4Δ::KAN rad52Δ::TRP* | This Study |
| YDV15.17 | *YDV1.17 exo1Δ::TRP sgs1Δ::KAN* | This Study |
| YDV16.17 | *YDV1.17 exo1Δ::TRP sgs1Δ::KAN mre11-H125N-URA3* | This Study |
| YDV17.17 | *YDV1.17 rad59Δ::TRP rad52Δ::URA3 rad51Δ::KAN* | This Study |
| YDV18.17 | *YDV1.17 rev3Δ::LEU2* | This Study |
| YDV19.17 | *YDV1.17 rad30Δ::TRP* | This Study |
| YDV20.17 | *YDV1.17 rev3Δ::LEU2 pol32Δ::KAN* | This Study |
| YDV21.17 | *YDV1.12 rad30Δ::TRP pol32Δ::KAN* | This Study |
| YDV22.17 | *YDV1.17 rev3Δ::LEU2 rad30Δ::TRP* | This Study |
| YDV23.17 | *YDV1.17 rev3Δ::LEU2 rad30Δ::TRP pol32Δ::KAN* | This Study |
| YDV60.18 | *JKM139 MAT****a****Z1::18bp microhomology::HPH* | This Study |
| YDV60.12 | *JKM139 MAT****a****Z1::12bp microhomology::HPH* | This Study |
| YDV100.17 | *JKM179 MATαZ1::HPH::17bp microhomology* | This Study |
| YDV100.13 | *JKM179 MATαZ1::HPH::13bp microhomology* | This Study |
| YDV200.17 | *SLY18 MATαZ1::HPH::17bp microhomology* | This Study |
| YDV200.13 | *SLY18 MATαZ1::HPH::13bp microhomology* | This Study |
| YDV300A | *JKM139 MAT****a****Z1::URA3* | This Study |
| YDV300B | *JKM179 MATαZ1::URA3::inverted MAT****a*** *cut-site* | This Study |
| YDV300.17 | *YDV300B MATαZ1::HPH::17bp microhomology* | This Study |
| YDV300.13 | *YDV300B MATαZ1::HPH::13bp microhomology* | This Study |
| YDV500.17 | *SLY60 MATαZ1::HPH::17bp microhomology to MATα* | This Study |
| YDV501A | *JKM179 ura3Δ0* | This Study |
| YDV501B | *JKM179 ura3-52::MAT****a****-cut-site URA3 AmpR 17bp microhomology to MATα and MAT****a*** | This Study |
| YDV501.17 | *YDV501B MATαZ1::HPH::17bp microhomology to MAT****a*** *and MATα* | This Study |
| YDV501B | *JKM179 ura3-52::MAT****a****-cut-site URA3 AmpR 17bp microhomology to MATα and MAT****a*** | This Study |
| YDV501.17 | *YDV501B MATαZ1::HPH::17bp microhomology to MAT****a*** *and MATα* | This Study |
| YDV515.17 | *YDV501.17 exo1Δ::TRP sgs1Δ::KAN* | This Study |

**References**

1. Ma JL, Kim EM, Haber JE, Lee SE (2003) Yeast Mre11 and Rad1 proteins define a Ku-independent mechanism to repair double-strand breaks lacking overlapping end sequences. Mol Cell Biol 23: 8820-8828.

2. Lee K, Zhang Y, Lee SE (2008) *Saccharomyces cerevisiae* ATM orthologue suppresses break-induced chromosome translocations. Nature 454: 543-546.

3. Shim EY, Chung WH, Nicolette ML, Zhang Y, Davis M, et al. (2010) *Saccharomyces cerevisiae* Mre11/Rad50/Xrs2 and Ku proteins regulate association of Exo1 and Dna2 with DNA breaks. EMBO J 29: 3370-3380.

4. Deem A, Keszthelyi A, Blackgrove T, Vayl A, Coffey B, et al. (2011) Break-induced replication is highly inaccurate. PLoS Biol 9: e1000594.

5. Toh GW, Sugawara N, Dong J, Toth R, Lee SE, et al. (2010) Mec1/Tel1-dependent phosphorylation of Slx4 stimulates Rad1-Rad10-dependent cleavage of non-homologous DNA tails. DNA Repair (Amst) 9: 718-726.

6. Vaze MB, Pellicioli A, Lee SE, Ira G, Liberi G, et al. (2002) Recovery from checkpoint-mediated arrest after repair of a double-strand break requires Srs2 helicase. Mol Cell 10: 373-385.
