# Supplementary material for: Microhomology Directs Diverse DNA Break Repair Pathways and Chromosomal Translocations
Source: PLoS Genet. 2012 Nov 8;8(11):e1003026. doi: 10.1371/journal.pgen.1003026 (PMC3493447; doi:10.1371/journal.pgen.1003026)
Supplement: Table S2 — Sequencing data. (DOC) [file pgen.1003026.s004.doc]

**Table S**2. Sequencing Data.

|  |  | **Hyg^S^ Surv.** | | **Hyg^R^ Surv.** | | |
| --- | --- | --- | --- | --- | --- | --- |
| **Strain Name** | **Strain Description** | **MH** | **Other MH^a^** | **NHEJ** | **Uncut** | **Other MH^a^** |
| YDV1.17 | 17 bp, 2 kb distance | 24/24 | 0/24 | NT | NT | NT |
| YDV1.16 | 16 bp, 2 kb distance | 23/24^b^ | 0/24 | NT | NT | NT |
| YDV1.15 | 15 bp, 2 kb distance | 24/24 | 0/24 | NT | NT | NT |
| YDV1.14 | 14 bp, 2 kb distance | 24/24 | 0/24 | NT | NT | NT |
| YDV1.13 | 13 bp, 2 kb distance | 23/24 | 1/24 | 23/24 | 0/24 | 1/24 |
| YDV3.12 | 12 bp, 2 kb distance, *yku70Δ* | 23/24^b^ | 0/24 | 0/8 | 6/8 | 2/8^g^ |
| YDV1.6 | 6 bp, 2 kb distance | 0/2 | 2/2 | NT | NT | NT |
| YDV3.6 | 6 bp, 2 kb distance, *yku70Δ* | NT | NT | 0/8 | 8/8 | 0/8 |
| YDV2.18 | 18 bp, 2 kb distance, *rad52Δ* | 46/46 | 0/46 | 12/14 | 2/14 | 0/14 |
| YDV2.17 | 17 bp, 2 kb distance, *rad52Δ* | 24/24 | 0/24 | NT | NT | NT |
| YDV2.16 | 16 bp, 2 kb distance, *rad52Δ* | 24/24 | 0/24 | NT | NT | NT |
| YDV2.15 | 15 bp, 2 kb distance, *rad52Δ* | 22/24^b^ | 1/24 | NT | NT | NT |
| YDV2.14 | 14 bp, 2 kb distance, *rad52Δ* | 18/19 | 1/19 | NT | NT | NT |
| YDV2.13 | 13 bp, 2 kb distance, *rad52Δ* | 15/16^b^ | 0/16 | NT | NT | NT |
| YDV2.12 | 12 bp, 2 kb distance, *rad52Δ* | 10/16^f^ | 1/16 | NT | NT | NT |
| YDV2.6 | 6 bp, 2 kb distance, *rad52Δ* | 0/3 | 3/3 | NT | NT | NT |
| YDV1.18.1MS | 18 bp: 1 mismatch, 2 kb distance | 24/24 | 0/24 | NT | NT | NT |
| YDV1.18.2CMS | 18 bp: 2 central mismatches, 2 kb distance | 23/24 | 1/24 | NT | NT | NT |
| YDV3.18.2LMS | 18 bp: 2 lateral mismatches, 2 kb distance, *yku70Δ* | 21/24^d^ | 0/24 | 0/24 | 17/24 | 7/24^h^ |
| YDV3.18.3MS | 18 bp: 3 mismatches, 2 kb distance, *yku70Δ* | 19/24^d^ | 2/24 | 0/16 | 16/16 | 0/16 |
| YDV4.18.1MS | 18 bp: 1 mismatch, 2 kb distance, *yku70Δrad52Δ* | 24/24 | 0/24 | 0/8 | 8/8 | 0/8 |
| YDV4.18.2CMS | 18 bp: 2 central mismatches, 2 kb distance, *yku70Δrad52Δ* | 22/24^c^ | 0/24 | 0/12 | 11/12 | 1/12 |
| YDV4.18.2LMS | 18 bp: 2 lateral mismatches, 2 kb distance, *yku70Δrad52Δ* | 19/24^f^ | 0/24 | 0/16 | 16/16 | 0/16 |
| YDV4.18.3MS | 18 bp: 3 mismatches, 2 kb distance, *yku70Δrad52Δ* | 19/24^e^ | 1/24 | NT | NT | NT |
| YDV500.17 | 2 breaks on separate chromosomes with 17 bp, 2 kb distance | 29/29 | 0/29 | NT | NT | NT |

Acronyms and Abbreviations: Hyg^S^, hygromycin sensitive; Hyg^R^, hygromycin resistant; Surv., survivors; MH, microhomology; NHEJ, non-homologous end-joining; NT, not tested; bp, base pairs; kb, kilobases

^a^Other MH: MH other than the given MH was found at the breakpoint junction, accompanied by deletions. When part of *HPH* was deleted, the resultant survivor was Hyg^S^.

^b^One breakpoint junction was not recovered by PCR, possibly due to larger flanking deletions at the break site.

^c^Two breakpoint junctions were not recovered by PCR. See description for ^b^.

^d^Three breakpoint junctions were not recovered by PCR. See description for ^b^.

^e^Four breakpoint junctions were not recovered by PCR. See description for ^b^.

^f^Five breakpoint junctions were not recovered by PCR. See description for ^b^.

^g^Both survivors had the same MH and deletion pattern at the breakpoint junction. This could possibly indicate a pre-existing DSB repair event.

^h^Six out of seven of these survivors had the same MH and deletion pattern at the breakpoint junction. This could possibly indicate a pre-existing DSB repair event.
