# Supplementary material for: Microhomology Directs Diverse DNA Break Repair Pathways and Chromosomal Translocations
Source: PLoS Genet. 2012 Nov 8;8(11):e1003026. doi: 10.1371/journal.pgen.1003026 (PMC3493447; doi:10.1371/journal.pgen.1003026)
Supplement: Table S3 — MHMR chromosomal translocations require resection. (DOC) [file pgen.1003026.s005.doc]

**Table S3. MHMR Chromosomal Translocations Require Resection.**

| Strains with microhomology on Chr. 3 and 5 | MHMR Survival Frequency | Fold Change | MHMR Translocation Frequency | Fold Change | NHEJ Survival Frequency | Fold Change | NHEJ Translocation Frequency | Fold Change |
| --- | --- | --- | --- | --- | --- | --- | --- | --- |
| 17 bp microhomology | 1.62 x 10^-2^ | -- | 0.64 x 10^-2^ | -- | 1.92 x 10^-5^ | -- | ND | -- |
| 17 bp microhomology *exo1Δ sgs1Δ* | 4.00 x 10^-6^ | 0.00025 | 1.60 x 10^-6^ | 0.00025 | 1.40 x 10^-4^ | 116 | 0.0574 x 10^-4^ | -- |

ND: none detected
