# Supplementary material for: Microhomology Directs Diverse DNA Break Repair Pathways and Chromosomal Translocations
Source: PLoS Genet. 2012 Nov 8;8(11):e1003026. doi: 10.1371/journal.pgen.1003026 (PMC3493447; doi:10.1371/journal.pgen.1003026)
Supplement: Table S4 — Melting temperatures. (DOC) [file pgen.1003026.s006.doc]

**Table S4**. Melting Temperatures.

| Length of Microhomology | Sequence | Melting Temperature (°C)^*^ |
| --- | --- | --- |
| 18 bp, MAT**a** | AGTTTCAGCTTTCCGCAA | 49.79 |
| 17 bp, MAT**a** | GTTTCAGCTTTCCGCAA | 47.41 |
| 16 bp, MAT**a** | TTTCAGCTTTCCGCAA | 45.59 |
| 15 bp, MAT**a** | TTCAGCTTTCCGCAA | 44.02 |
| 14 bp, MAT**a** | TCAGCTTTCCGCAA | 42.25 |
| 13 bp, MAT**a** | CAGCTTTCCGCAA | 39.37 |
| 12 bp, MAT**a** | AGCTTTCCGCAA | 35.22 |
| 6 bp, MAT**a** | CCGCAA | -- |

^*^Calculated using the “Nearest Neighbor” method, using the website:

http://www.basic.northwestern.edu/biotools/oligocalc.html#helpthermo
